# 3D-QSAR, Scaffold Hopping, Virtual Screening, and Molecular Dynamics Simulations of Pyridin-2-one as mIDH1 Inhibitors

**DOI:** 10.3390/ijms25137434

**Published:** 2024-07-06

**Authors:** Yifan Wang, Shunjiang Jia, Fan Wang, Ruizhe Jiang, Xiaodan Yin, Shuo Wang, Ruyi Jin, Hui Guo, Yuping Tang, Yuwei Wang

**Affiliations:** 1 College of Pharmacy, Shaanxi University of Chinese Medicine, Shiji Ave, Xi’an-Xianyang New Economic Zone, Xianyang 712046, China; 222031712627@email.sntcm.edu.cn (Y.W.); 520051301416@email.sntcm.edu.cn (S.J.); jinruyi335588@163.com (R.J.); guohui@sntcm.edu.cn (H.G.); yupingtang@sntcm.edu.cn (Y.T.); 2 Second Clinical Medical College, Shaanxi University of Chinese Medicine, Shiji Ave, Xi’an-Xianyang New Economic Zone, Xianyang 712046, China; 520040101433@email.sntcm.edu.cn (F.W.); 522030106504@email.sntcm.edu.cn (R.J.); 3 State Key Laboratory of Quality Research in Chinese Medicine, Macau University of Science and Technology, Macau 999078, China; yinxd14@lzu.edu.cn; 4 College of Pharmacy, Lanzhou University, Lanzhou 730000, China

**Keywords:** IDH1, CoMFA, CoMSIA, scaffold hopping, molecular dynamics simulations

## Abstract

Isocitrate dehydrogenase 1 (IDH1) is a necessary enzyme for cellular respiration in the tricarboxylic acid cycle. Mutant isocitrate dehydrogenase 1 (mIDH1) has been detected overexpressed in a variety of cancers. mIDH1 inhibitor ivosidenib (AG-120) was only approved by the Food and Drug Administration (FDA) for marketing, nevertheless, a range of resistance has been frequently reported. In this study, several mIDH1 inhibitors with the common backbone pyridin-2-one were explored using the three-dimensional structure–activity relationship (3D-QSAR), scaffold hopping, absorption, distribution, metabolism, excretion (ADME) prediction, and molecular dynamics (MD) simulations. Comparative molecular field analysis (CoMFA, R^2^ = 0.980, Q^2^ = 0.765) and comparative molecular similarity index analysis (CoMSIA, R^2^ = 0.997, Q^2^ = 0.770) were used to build 3D-QSAR models, which yielded notably decent predictive ability. A series of novel structures was designed through scaffold hopping. The predicted pIC_50_ values of C3, C6, and C9 were higher in the model of 3D-QSAR. Additionally, MD simulations culminated in the identification of potent mIDH1 inhibitors, exhibiting strong binding interactions, while the analyzed parameters were free energy landscape (FEL), radius of gyration (Rg), solvent accessible surface area (SASA), and polar surface area (PSA). Binding free energy demonstrated that C2 exhibited the highest binding free energy with IDH1, which was −93.25 ± 5.20 kcal/mol. This research offers theoretical guidance for the rational design of novel mIDH1 inhibitors.

## 1. Introduction

Targeting tumor metabolism is considered a highly promising new strategy for anti-tumor treatment and has become a hot area in the advancement of new anti-tumor drugs in recent years [1]. IDH1 is a pivotal rate-limiting enzyme in cellular glycolysis, which is one of the most widely mutated metabolic enzymes in human tumors. mIDH1 has been discovered in a variety of tumors, including cholangiocarcinoma, chondrosarcoma, glioma, and acute myeloid leukemia (AML), and so on [2]. Studies have shown that mIDH1 acquires a new catalytic function, catalyzing the conversion of α-ketoglutarate (α-KG) to 2-hydroxyglutarate (2-HG) [3]. 2-HG is a recognized cancer metabolite that can promote the progression of a malignant tumor through diffident mechanisms including influencing the levels of histone methylation, thus driving the occurrence of a malignant tumor [4].

IDH1 plays an essential role in the regulation of tumor, leading to a change of enzymic activity, which in turn affects the cycle of citric acid and metabolic pathways within cells, resulting in the accumulation of metabolites such as lactate and pyruvate [5]. The accumulation of metabolite can react on the tumor microenvironment, causing alteration in DNA and protein modifications, thereby influencing proliferation, differentiation, cell apoptosis, and other biological processes [6]. With these mechanisms, IDH1 is regarded as a subject of great interest in cancer research. mIDH1 is connected with the development of numerous cancers, making it a focal point in cancer research [7].

Due to the significant role of IDH1 in tumor progression, many researchers have become dedicated to discover and identify compounds that can inhibit the activity of mIDH1. In July 2018, AG-120 was approved by the FDA for the treatment of patients with relapsed or refractory AML carrying IDH1 susceptible mutations [8]. AG-120 is currently the only FDA-approved targeted therapy for AML patients with IDH1^R132H^ and IDH1^R132C^ [9]. Furthermore, plenty of effective mIDH1 inhibitors targeting both IDH1^R132H^ and IDH1^R132C^ were reported, exhibiting nanomolar activity against mIDH1 and reducing the intracellular product 2-HG catalyzed by mIDH1 [10]. Accordingly, the design and development of novel mIDH1 inhibitors targeting mIDH1 can be confirmed as a useful approach for cancer therapy [11].

QSAR is a mathematical and statistical method to study quantitatively the interaction of organic small molecules with biomolecules and their structure–activity relationship by means of physicochemical or structural parameters of molecules [12]. 3D-QSAR introduces three-dimensional structural information of molecules for studies of structure–activity relationship, which can indirectly respond to the characteristics of non-bonded interaction between molecules and biomolecules in the process of interaction [13,14]. The interaction of drug and the acceptor is achieved in a reversible way, such as by van der Waals force, electrostatic gravity, hydrogen bonding, hydrophobic interaction, etc. The method uses powerful chemical computational techniques to calculate the activity of ligands using their three-dimensional properties [15]. Although various experimental error factors in designing new drugs cannot be completely ignored, it is definitely possible to select the most active drug to reduce the number of compounds to be synthesized. Therefore, this method has become a valuable predictive tool in the design of the required chemicals [16]. The method of 3D-QSAR was rapidly developed in the last decade [17]. CoMFA and CoMSIA were used to build a model as the two most popular methods in order to clarify the relationships of structure–activity and offer guidance for optimization [18,19,20].

**Figure 1:**
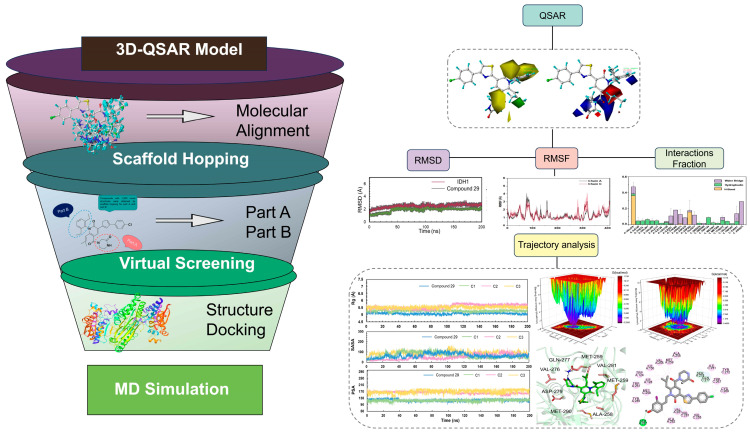
The workflow of virtual screening for IDH1 inhibitor.

Herein, a total of 47 compounds were collected to construct the models of 3D-QSAR. According to 3D-QSAR models, a series of compounds was designed via scaffold hopping, and then 100 compounds with the best docking scores were identified via virtual screening. Through MD simulations, the binding mode, binding stability, and binding free energy of the C1, C2, C3 compounds with the best docking scores were explored, and it was found that C1, C2, C3 had higher binding free energy than the positive control compound **29**. The workflow of the whole work is shown in Figure 1. These series of theoretical and computational studies laid a theoretical foundation for designing and optimizing the mIDH1 inhibitors. Many molecules with high predicted activity were designed to contribute valuable theoretical insights for the prediction of activity and structural modification of pyridin-2-one-based targeted mIDH1 inhibitors.

## 2. Results

### 2.1. Data Sets and Molecular Alignment

**Figure 2:**
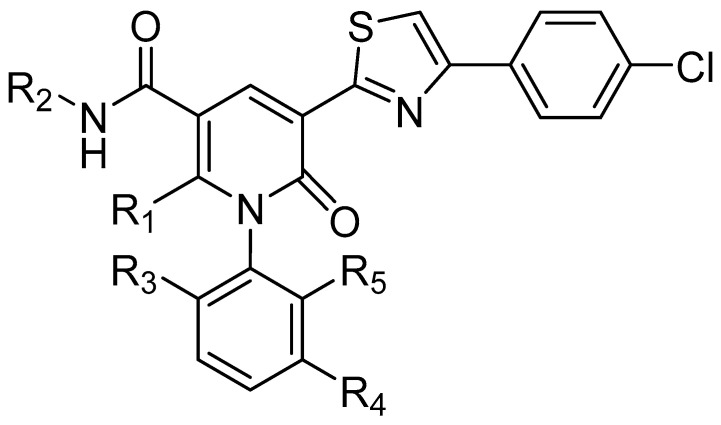
Common skeleton of a series of pyridin-2-one compounds.

All 2H 1λ^2^ pyridin-2-ones 7,7-dimethyl-7,8-dihydro-2H-1λ^2^-quinoline-2,5(6H)-diones and their activities of associated inhibition were obtained. Figure 2 shows the common skeleton of these 47 compounds. Among them, the training set included 38 compounds and the test set included nine compounds. The structures of all compounds and their biological activities are shown in Table 1. It was observed that the best inhibitory activity value of the enzyme was compound **29** with an activity value of 0.035 μM; however, the compound with the worst activity value was compound **11** with an activity value of 4.200 μM.

**Figure 3:**
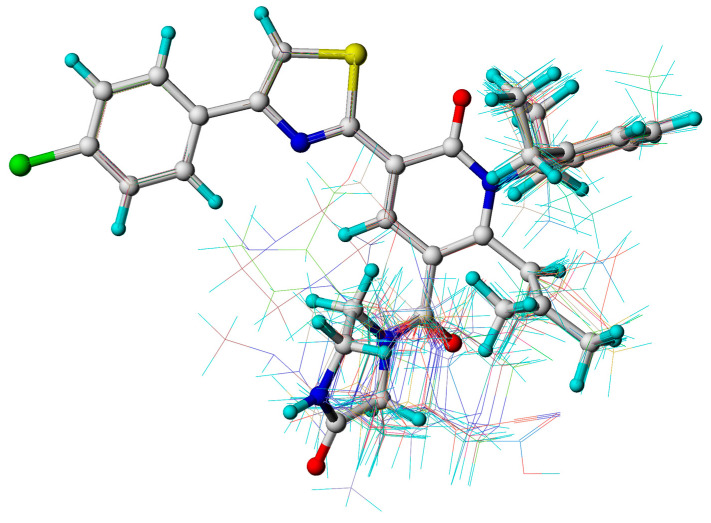
Structural alignment of all the molecules in the training set, based on the common skeleton of compound **29**.

Based on the structure and inhibitory activity of all molecules, the compounds in the training set were aligned using the most active compound **29** as a template molecule. In Figure 3, the common backbones of all the molecules overlap.

### 2.2. CoMFA and CoMSIA

As a whole, R^2^ > 0.7, Q^2^ > 0.5 are essential for a good model [21]. Q^2^ and R^2^ were obtained to assess the predictive power of the 3D-QSAR model. The CoMFA model showed that the values of Q^2^, N, SEE, R^2^, R^2^_pred_, and F are, respectively, 0.765, 6, 0.091, 0.980, 0.943, and 253.629. The outcomes illustrated that the model of CoMFA expresses decent predictive ability. The contribution of the steric field is 58.2%, and the electrostatic field is 41.8%, demonstrating that the effects of steric and electrostatic fields on the biological activity of the compounds are similar. The calculated results validate that the prediction is reliable.

The parameters obtained from several CoMSIA fields are different. By combining various fields, it was confirmed that CoMSIA-SEH is the best optimal model (Table 2). The CoMSIA model presented that the values of Q^2^, N, SEE, R^2^, R^2^_pred_, and F are partly 0.77, 10, 0.04, 0.997, 0.980, and 800.063. In this model, the contribution of the steric field, electrostatic field, and hydrophobic field is 22.5%, 44.4%, and 33.1%, separately. The consequence is that the electrostatic field has a greater effect on the bioactivity of the mIDH1 inhibitors. The parameters of statistics of CoMFA and CoMSIA are presented in Table 3.

**Figure 4:**
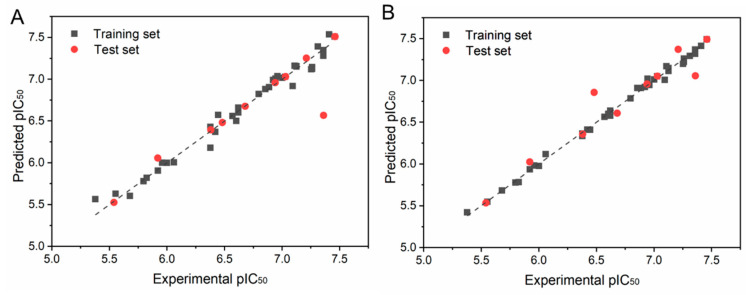
Scatter plot of experimental and predicted bioactivity values (pIC_50_) of the CoMFA (**A**) and CoMSIA models (**B**), respectively.

Table 4 shows the experimental and predicted values of the biological activity of the training and the test set of CoMFA and CoMSIA. In CoMFA and COMSIA, the predicted values of compounds were dramatically close to the experimental values, and most of the differences between the experimental values and the predicted values were in the range of 0.1. However, compound **17** had a large difference in the CoMFA, while compound **13**, compound **17**, and compound **37** had a larger difference in CoMSIA, which may be due to the specificity of their structures. The scatter plot of the experimental and predicted values is shown in Figure 4. It can be noticed from Figure 4 that the experimental and predicted bioactivity values of the 47 compounds are basically distributed about the Y = X equation, demonstrating that decent predictive power exists in the model.

### 2.3. The Analysis of 3D Contour Maps

**Figure 5:**
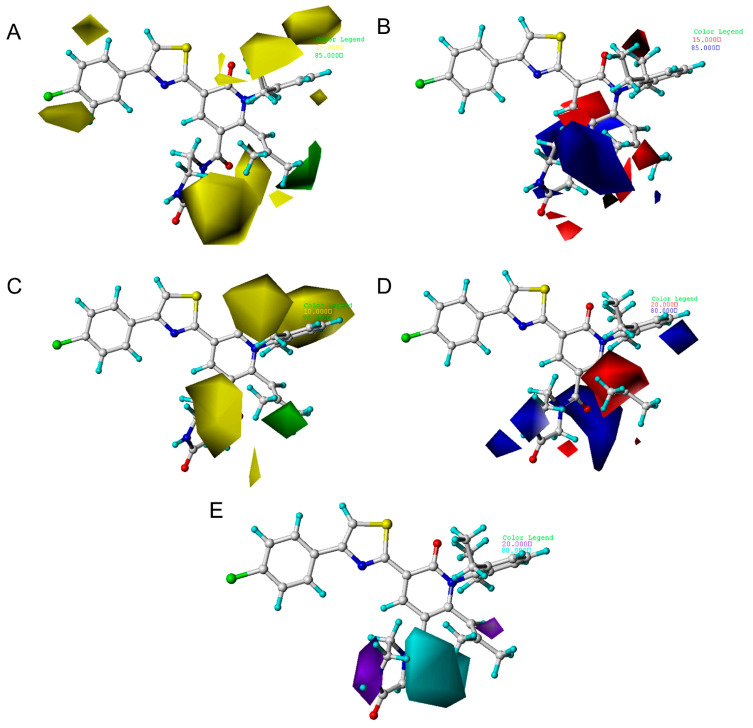
Contour maps of the CoMFA and CoMSIA models using compound **29** as a reference. (**A**) The steric field contour map of the CoMFA model. (**B**) The electrostatic field contour map of the CoMFA model. (**C**) The steric field contour map of the CoMSIA model. (**D**) The electrostatic field contour map of the CoMSIA model. (**E**) The hydrophobic field contour map of the CoMSIA model.

The key features contributing to the binding affinity of the ligand were identified. The CoMFA and CoMSIA were graphically interpreted using maps of the STDEV*COEFF type. The steric and electrostatic contour plots of CoMFA, respectively, are displayed in Figure 5. In Figure 5A, the green contours (85% contribution) and yellow contours (15% contribution) separately indicate the steric favorable and non-favorable regions of activity. In Figure 5B, the blue contour (85% contribution) describes the region where positive electrostatic field is favored. The red contour (15% contribution) shows the region where negative electrostatic field is favored. Compound **29** (pIC_50_ = 7.46) with the best biological activity was used as a reference for the model of CoMFA and CoMISA.

Figure 5A shows the steric field of CoMFA, where the green region indicates that a large group is favorable for the enhancement of the biological activity, while the yellow region is the opposite. As seen in Figure 5A, the R1 substituent is wrapped in green, which proves that the introduction of a large group in this region can improve the biological activity. Compared with compound **20** (pIC_50_ = 6.62) and compound **21** (pIC_50_ = 6.13), the two compounds differ only in the R1 part. Compound **21** connects to tetrahydrofuran at the R1 position, so the value of enzyme activity is much better than compound **20**. Comparing compound **15** and compound **16**, the substitution of the R1 site is different—R1 of compound **15** is substituted by isopropyl and R1 of compound **16** is substituted by isobutyl—and the activity of compound **16** (pIC_50_ = 6.96) is greater than that of compound **15** (pIC_50_ = 6.38). The R5 substituent had a much yellower color block, which proved that the introduction of small groups, such as hydrogen atoms, can be beneficial for improving activity. For example, the activity of compound **39** (pIC_50_ = 6.57) was less than that of compound **38** (pIC_50_ = 7.25) after the introduction of a methyl group.

The contour maps for the electrostatic field of CoMFA are displayed in Figure 5B. The red region represents that the negatively charged group can be conducive to the enhancement of activity. The blue region indicates that the positively charged group is favorable to increase activity. It can be observed that there was a large blue area near the R2 substituents, indicating that the presence of positively charged groups is favorable for biological activity enhancement. In comparison with compound **7** and compound **8**, the R2 site of compound **7** is connected with the positive charge group CH_2_CH_2_NH_2_, and its enzyme inhibitory activity is 0.38 μM, while that of compound **8** is 0.87 μM. A blue area near the piperazine substituent indicates that the substituent is positively charged in favor of enhanced biological activity. A large blue area near R1 represents that the introduction of a positively charged group has an enhanced effect on biological activity. For example, compound **17** (pIC_50_ = 7.36) has better biological activity than compound **20** (pIC_50_ = 6.62). This suggests that the introduced positively charged group may have favorable interactions with the surrounding residues.

The steric field of the CoMSIA model is similar to the electrostatic field model (Figure 5). Figure 5E shows the hydrophobic field in CoMSIA. The cyan area indicates that the hydrophobic group is favorable and easily binds to other molecules to form hydrophobic interactions, indicating that the hydrophobic group is vital to inhibit activity, while the white color shows the opposite. The large purple area at the R1 substituent indicates that this may be an unfavorable position for the hydrophobic group. The large cyan area wrapped around R2 is a favorable position for the hydrophobic group, which may form hydrophobic interactions with the mIDH1 enzyme at the binding site. On comparing compounds **12** and **15**, the substitution site of R1 is different; R1 of compound **12** is substituted by the hydrophobic group methyl, so the biological activity of compound **12** (pIC_50_ = 6.68) is higher than that of compound **15** (pIC_50_ = 6.38).

**Figure 6:**
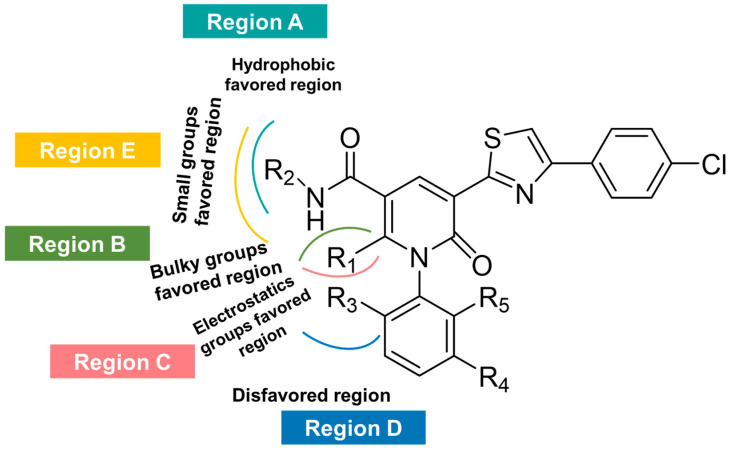
Structure–activity relationship diagram of mIDH1 inhibitors.

According to the results of the analysis of the model, the QSAR of pyridin-2-one derivatives are displayed in Figure 6. The introduction of a hydrophobic group in Region A is helpful to improve the activity of compounds, such as the aryl, ester, ether, etc. The introduction of substitutes with a large space in Region B is conducive to the activity of the compounds, e.g., biphenyl or p-cyclohexylbenzene. For example, in compound **13** (pIC_50_ = 5.54) and compound **15** (pIC_50_ = 6.38), the R1 site of compound **15** is occupied by the large isopropyl group, so the enzyme inhibitory activity is better. Region C is a favored region of electrostatics groups. Region D is a disfavored region. Region E is a favored region for small group. For instance, between compound **1** (pIC_50_ = 5.82) and compound **3** (pIC_50_ = 5.55), the R2 site of compound **1** is occupied by small methyl groups, so the enzyme inhibitory activity is poor, whereas the R2 of compound **3** is occupied by isopropyl groups.

### 2.4. Scaffold Hopping and Virtual Screening

**Figure 7:**
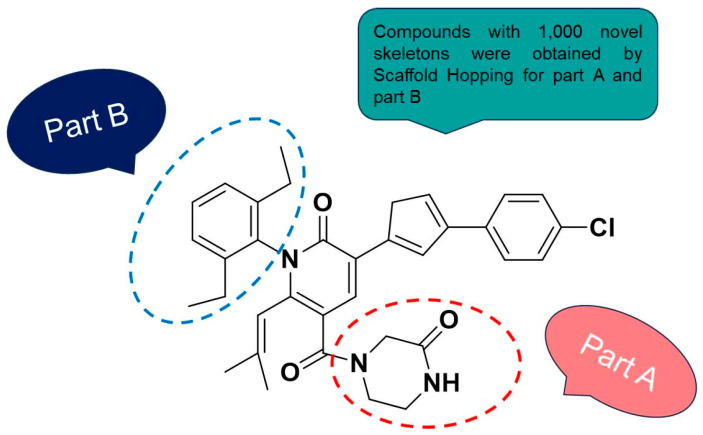
The 2D structure of compound **29**.

Scaffold hopping was performed on compound **29** with the best biological activity, where part A and the part B, respectively, were used for scaffold hopping (Figure 7). A total of 1000 compounds were obtained through scaffold hopping to obtain a virtual database, which was virtually screened based on docking to obtain 100 new structures with the best docking score, and all of these 100 compounds had docking scores greater than compound **29**.

Based on the consequence of QSAR, a string of compounds with the pyridin-2-one skeleton were designed as promising mIDH1 inhibitors by introducing new substituents at different positions of compound **29**. In Table 5, the top nine compounds with the best docking score were listed with structures and pIC_50_ for predicting. As a result of the scaffold hopping and virtual screening, it was observed that the red substituents of the top nine compounds are occupied by piperazin-2-one, which suggests that the introduction of this substituent may be able to form a hydrogen bond at this site. The introduction of some small groups at R2 increases the activity, and the predicted activity of CoMFA of C6 is better than that of other small molecules. The introduction of some large groups and hydrophobic groups at R3, R4, R5 is favorable to increase the activity; alkyl, halogen, and long-chain hydrocarbons are typical hydrophobic groups, so the predicted activity of C1, C3, C6, C9 is better than that of other small molecules as a whole. In addition, the synthetic accessibility score (SA score) was evaluated. The synthesis difficulty of small molecules is evaluated with values ranging from 1 to 10. The closer to 1, the easier is the synthesis; the closer to 10, the more difficult is the synthesis is. The SA scores of the top nine designed compounds were all around 3, indicating that the difficulty of synthesis of this series of compounds was similar.

### 2.5. ADME Prediction for Designed Compounds

**Figure 8:**
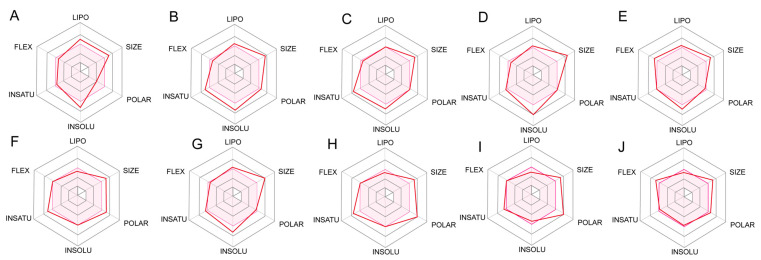
The pharmacokinetics of compound **29** (**A**), C1 (**B**), C2 (**C**), C3 (**D**), C4 (**E**), C5 (**F**), C6 (**G**), C7 (**H**), C8 (**I**), C9 (**J**) obtained from SwissADME (The pink area represents the optimal range for each property).

The predicted ADME of the top 7 compounds with the best docking score and compound **29** are shown in Figure 8. C1, C2, C3, C4, C6 have better lipophilicity (LIPO), which represents generally easier passage through the lipid bilayer of the cell membrane and therefore higher uptake rates. However, the poorer LIPO of C5, C7 is speculated to be probably due to the fact that the O atom above the substituent of the part A is directly attached to the pyridine ring, and as a result showing poor LIPO. These seven compounds are similar in their polarity and size due to their similar structure. C3 and C8 also have poor polarity.

Understanding and optimizing the insolubility (INSOLU) of a drug candidate is critical to its clinical effectiveness and successful development. Drugs with high solubility dissolve rapidly in the gastrointestinal tract, forming a state of solution that facilitates the absorption of drug. C5, C7, C9, C10 are more soluble and usually require lower doses, thus reducing the risk of adverse reactions and toxicity. Insaturation (INSATU) refers primarily to the proportion of the molecular structure that is monounsaturated. C3, C4, C5, C6, C8, C9 are more saturated. More saturated molecules are generally more stable than unsaturated molecules because the presence of double and triple bonds may make the molecule more susceptible to oxidation or other chemical reactions that can lead to degradation.

### 2.6. Molecular Dynamics Simulations

MD simulations is regarded as a complementary strategy for identifying the molecular interactions between ligands and proteins [22]. To explore the stability of ligand-receptor complexes for C1, C2, C3 with the best docking scores, compound **29** was used as a positive control to validate the compound with good binding affinities.

**Figure 9:**
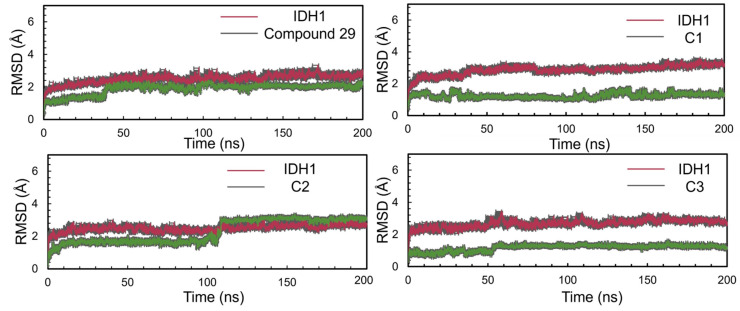
RMSD of the protein–ligand interaction throughout the 200 ns simulations.

As shown in Figure 9, the Root mean squared deviation (RMSD)of compound **29**, C1, C2, C3 eventually reaches a steady state while fluctuation is less than 2 Å. For compound **29**, from 39 ns, there was an increase of RMSD from about 1.28 Å to about 2.0 Å. Then the equilibrium was maintained at about 2.04 Å. Throughout the simulation, the fluctuation of backbone was small and was overall stable. For C1, before the first 30 ns, the RMSD of the ligand was unstable. Over 30–100 ns, the RMSD value of the ligand stabilized at around 1.15 Å and was more stable towards the end of the MD simulations. For C2, a large increase in RMSD values to 3.12 Å after 108 ns was noticed; the values of RMSD were maintained at about 3.03 Å and remained stable until the end of the simulations. In the case of C3, the RMSD of ligand increased to 1.20 Å until it stabilized after 50 ns. The overall backbone did not fluctuate much during MD simulations. It can be seen that the IDH1 protein binds better to compound **29**, C1, C2, and C3, and none of the backbones displayed much deviation during the MD simulations of 200 ns.

**Figure 10:**
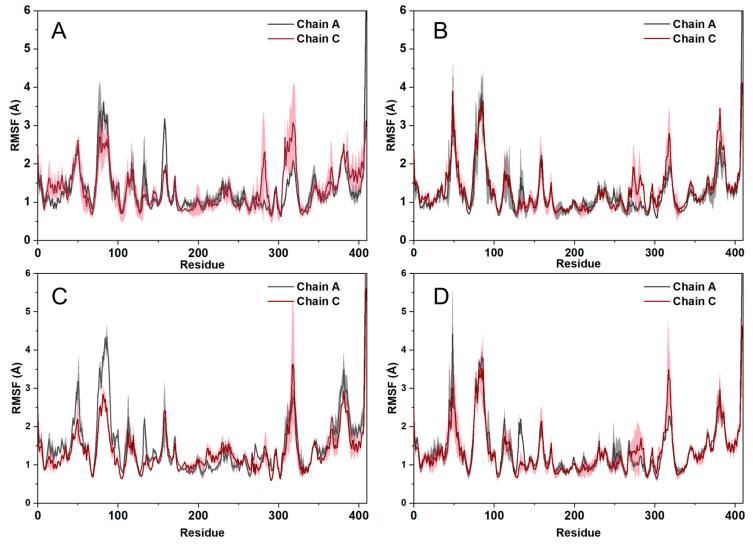
RMSF of compound **29** (**A**), C1 (**B**), C2 (**C**), C3 (**D**) with IDH1 protein.

The Root mean square fluctuation (RMSF)values of residues in the complex reflect the change in the position of each atom. For different atoms, a higher RMSF value implies a greater position change and greater flexibility in the binding pocket during the simulation [23,24]. The result of the RMSF of compound **29**, C1, C2, and C3 in IDH1 protein is shown in Figure 10. C3 maintained equilibrium during MD simulations, without large fluctuations whereas the other two compounds C1 and C2 varied with little fluctuations after a certain period of time due to their rigid structure. As a result, the majority of protein residues in these complexes exhibited RMSF values of less than 2 Å. The binding trend of C1, C2, and C3 in IDH1 protein was similar to that of compound **29**.

**Figure 11:**
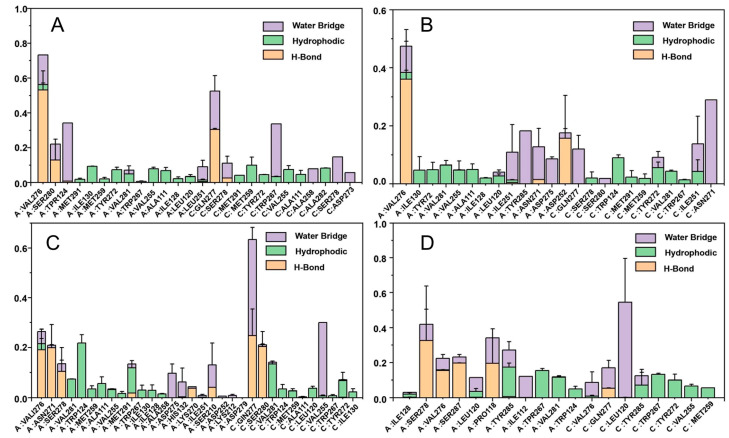
Contribution of individual active site residues of compound **29** (**A**), C1 (**B**), C2 (**C**), C3 (**D**) to inhibitor binding in IDH1 complexes present during MD simulations.

The histogram of protein–ligand interactions is presented in Figure 11. For compound **29**, VAL276, SER278, GLN277 are key residues that form hydrogen bonding interaction. VAL276 is also a vital residue that forms a hydrogen bonding interaction with C1. With reference to C2, ASN271, VAL276, GLN277, and SER280 may form hydrogen bonding, nevertheless the percentage formation is small. In the case of C3, PRO118 and SER287 may form hydrogen bonding. From this, it is surmised that residue GLN277 and VAL276 may play an essential role during the binding of the IDH1 protein with the ligand, while the binding of these two residues enhances the binding force between the protein and the ligand, which may help to maintain the stability of its structure. Through the formation of hydrogen bonds, a specific steric conformation can be created between protein and ligand, thus affecting their functions and interactions. This helps to ensure specific binding between protein and ligand, and is of great significance for signaling, regulation of metabolism, and other biological processes in organisms.

### 2.7. The Analysis of Free Energy Landscape, Radius of Gyration, Solvent Accessible Surface Area, and Polar Surface Area

**Figure 12:**
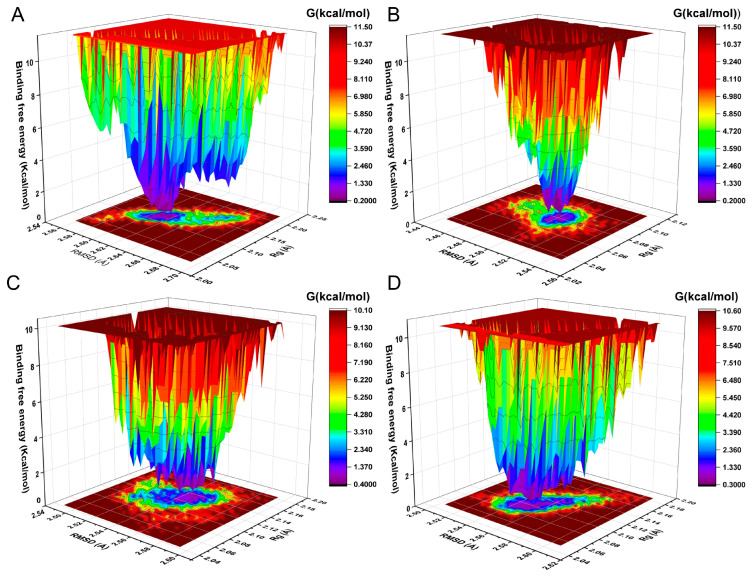
Free energy landscapes of the compound **29** (**A**), C1 **(B**), C2 (**C**), C3 (**D**).

The FEL of achieving global minima of backbone atoms of proteins concerning RMSD and Rg is displayed in Figure 12. In FEL, the mIDH1 systems achieved the lowest energy conformation. The conformational transition within each complex is delineated by a subspace, indicating that these small molecule inhibitors bind to the protein through different binding modes, resulting in minimal binding effects.

**Figure 13:**
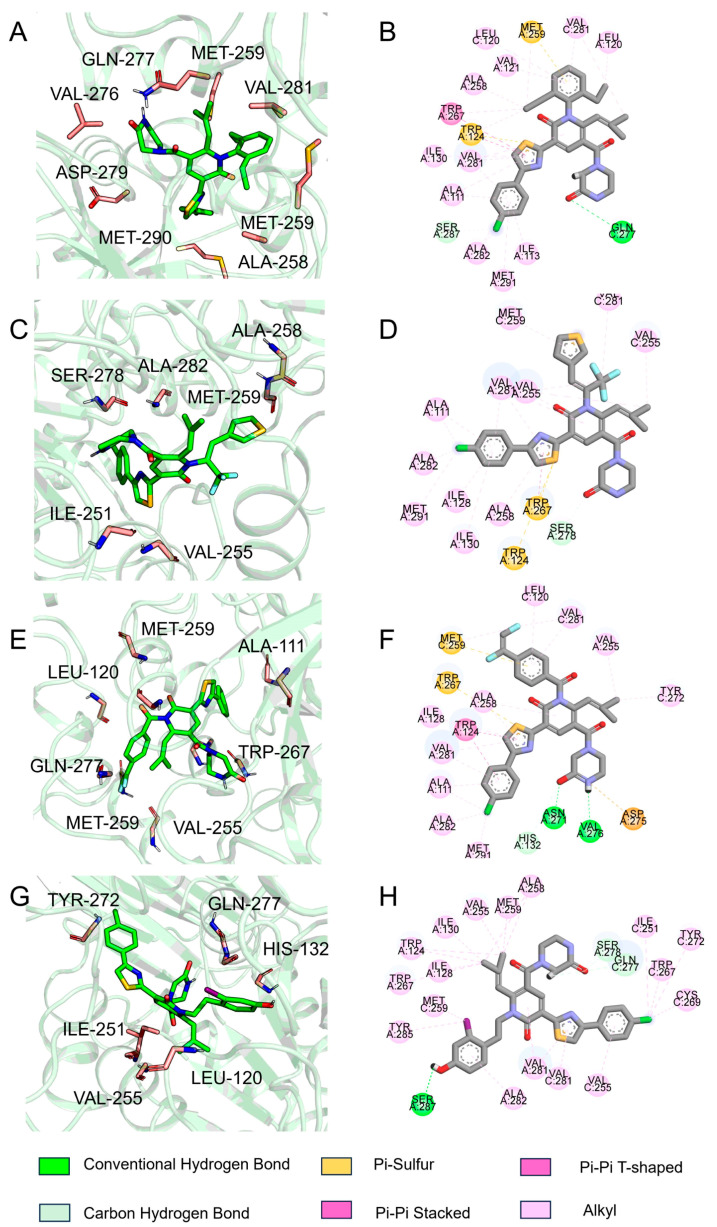
3D Binding model diagram of compound **29** (**A**), C1 (**C**), C2 (**E**), C3 (**G**), 2D Binding model diagram of compound **29** (**B**), C1 (**D**), C2 (**F**), C3 (**H**).

The conformations of the lowest energy of FEL were extracted to analyze the binding modes. The representative conformations of compound **29**, C1, C2, and C3 are displayed in Figure 13. For compound **29**, GLN277 formed a conventional hydrogen bond with the double bond O on piperazine. The thiazole ring of compound **29** formed π-π stacked with LEU120. The amino acid residues of the binding cavity that interacted with C1 were ALA111, VAL281, ALA258, MET291, etc. TRP267 and MET124 formed π-sulfur with thiophene in C1. About C2, ASN271 formed conventional hydrogen bonds with the atom of O on the side chain of the pyridin-2-one. VAL276 formed a conventional hydrogen bond with the atom of S on the pyridin-2-one. For C3, SER287 formed a conventional hydrogen bond with the hydroxyl group on the benzene ring. Alkyl plays an important role in the binding of C3 to IDH1 proteins. This is consistent with the results of analysis of the binding modes throughout the whole process of MD simulation.

**Figure 14:**
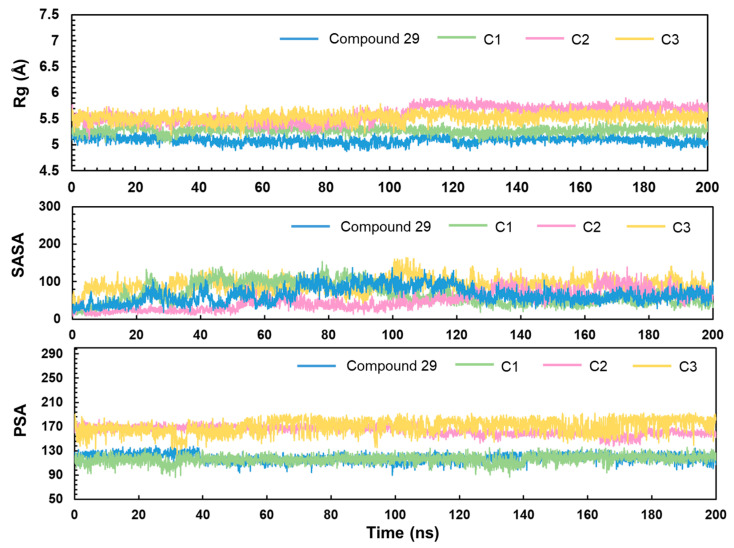
The analysis of Rg, SASA, and PSA of compound **29**, C1, C2, C3.

In Figure 14, Rg values of compound **29**, C1, C2, and C3 during the MD simulations were calculated to study the tightness of ligand binding throughout the MD simulations. The four compounds are stably bound with the Rg values at 5–5.5 Å in MD simulations. For drug design, assessment of SASA at the binding site of a candidate to protein helps to optimize the molecule to improve its affinity and selectivity. In Figure 14, SASA of the ligands appears to change when the ligand first binds to the protein due to the ligand maybe obscuring the surface of some amino acids of the protein, which makes the SASA of the ligand increase and that of the protein decrease. For example, the SASA of compound **29**, C2, and C3 started to have a significant increase, and the fluctuation stabilized within 140–200 ns. In general, the overall fluctuation is relatively stable during MD simulation. PSA is the surface area occupied by all polar atoms on the surface of a molecule (oxygen and nitrogen, and the hydrogen atoms attached to oxygen/nitrogen), which is used to assess the polar nature of a molecule and its ability to interact with biological membranes. Ligands with lower PSA values are more likely to cross the lipid bilayer by passive diffusion. The PSA values of compound **29** and C1 are around 120 Å^2^, which indicates that the ligands have some ability to pass through the cell membrane. It may be capable of crossing the cell membrane by passive diffusion. However, C2 and C3 are less capable of crossing the cell membrane by passive diffusion and may require active transport or other special channels to cross the cell membrane efficiently.

### 2.8. Binding Free Energy Calculation

To calculate the binding affinity between the ligands and the binding pockets, the molecular mechanics/generalized Born surface area (MM-GBSA) was utilized to calculate the ΔG_bind_ in each system [25,26]. A total of 1000 snapshots were extracted from the 3000–4000 snapshots to calculate the binding free energy. The result is shown in Table 6. Among these compounds, C2 expressed the highest binding free energy in IDH1, which is −93.25 ± 5.20 kcal/mol. C1 has a binding energy of −76.34 ± 4.67 kcal/mol and C3 has a binding free energy of −63.77 ± 3.18 kcal/mol. Though the calculation of binding free energy, it was concluded that the binding free energy of C1, C2, C3 in the IDH1 complex is higher than in compound **29**.

## 3. Discussion

To the best of our knowledge, 47 compounds with pyridin-2-one for detection to be active were collected to construct the modeling of this series of structures in 3D-QSAR, based on the known IC_50_ values. When the model occurred with r^2^ > 0.5 and q^2^ > 0.5, the 3D-QSAR model of construction was reliable and had good predictive ability [27]. When the model occurred with q^2^ > 0.7, the 3D-QSAR model of construction is reliable and relatively with ability of excellent prediction [28]. Herein, QSAR models were successfully constructed with good predictive ability. The best CoMFA model showed a cross-validation coefficient Q^2^ of 0.765. The best CoMSIA model (including steric, electrostatic, and hydrophobic fields) had a Q^2^ value of 0.770 and an optimized component of 10.

The scaffold hopping is a strategy for the discovery of new compounds by altering specific segments of a molecule to obtain a novel chemical structure, usually starting from a known active compound [29]. Compound **29** as a template molecule was selected for the scaffold hopping, which was performed by two parts separately for hopping to obtain 1000 new compounds. Then, 1000 compounds were subjected to virtual screening based-docking to extract the top 100 compounds with the best docking scores. Fortunately, the 100 compounds which were extracted through scaffold hopping had better scores than compound **29** with known activity, suggesting that it is possible that the activity of these 100 new compounds is even better than compound **29**. From these 100 compounds, the top 7 compounds with the best docking scores were selected for subsequent analysis.

For the purpose to explore initially LIPO, INSOLU, INSATU and other relevant parameters of the compounds, pharmacokinetic predictions of the top nine compounds obtained from the screening were performed using the online website SwissADME. Ultimately, it was found that these top nine compounds are very similar in terms of lipid solubility as well as molecular weight, due to the great similarity in the skeleton as well as the substituent part of the structure. Therefore, the pharmacokinetic properties of these compounds are similar, and all of them present decent drug-likeness. Especially, C1, C3 and C5 are better in terms of solubility and saturation. However, the difficulty of synthesizing this series of compounds along with their specific properties needs to be investigated.

Although 3D-QSAR and scaffold hopping were used to obtain a series of inhibitors with decent docking scores, the specific binding modes of the small molecules to the protein receptor are still unclear. Protein–ligand complexes with good binding have the potential to show potent inhibitory effects in vivo or in vitro after being investigated as drugs. The exploration of binding free energy and binding interactions is an important part of computational chemistry. Therefore, the top three compounds with the best docking scores were subjected to MD for 200 ns to explore the interaction of the representative compounds with the best scores. From the analysis of MD, it was found that during the process of simulation, the binding of the protein and ligand was stable, and there was not much fluctuation in the process of 200 ns, while the value of RMSD was relatively small overall. In the analysis of interactions, it was also found that VAL276 is a vital residue for the formation of hydrogen bonds in C1. ASN271, VAL276, GLN277, and SER280 formed hydrogen bonds in the simulation process of C2. In the binding of C3, PRO118, HIS132, ILE130, ASP275, TYR285, and VAL276 formed hydrogen bond interactions. The formation of hydrogen bonds can affect the conformation and stability of molecules and is helpful for the interaction between drugs and targeting proteins. Hydrogen bonds can be used to enhance the selectivity, affinity, and efficacy of drugs. This effect helps to improve the bioavailability and pharmacological characteristics of drugs, thereby enhancing the therapeutic effect of drugs.

FEL is a significant tool used to analyze the changes of energy in molecular systems in different conformational spaces, which can be beneficial for understanding the MD behavior, stability, and functional mechanisms of the system [30]. From the FEL, the conformation with the lowest energy of the system was obtained for analyzing the binding modes in each IDH1 complex system. It was observed that GLN277 formed a conventional hydrogen bond with the double bond O on piperazine for compound **29**. TRP267 and MET124 formed π-sulfur with thiophene in C1. ASN271 formed conventional hydrogen bonds with the atom of O on the side chain of the pyridin-2-one of C2 in a complex with IDH1, while VAL276 formed a conventional hydrogen bond with the atom of S on the pyridin-2-one. SER287 formed a conventional hydrogen bond with the hydroxyl group on the benzene ring for C3. To begin with, the interactions of some residues with the highest binding frequency and the greatest role were obtained from the analysis of the process of MD simulation. For the process of C2, complexed with IDH1 protein, both ASN271 and VAL276 on the chain A formed hydrogen bonds. Furthermore, the binding model of the conformation with the lowest energy frame showed that ASN271 and VAL276 on the A chain formed hydrogen bonds too in the FEL analysis. In the interaction of C1 binding to IDH1 protein, VAL276 formed hydrogen bonds. However, no hydrogen bonding interactions were shown in the binding model of the frames with the lowest energy extracted by FEL, which is due to the fact that VAL276 plays a role with a frequency of less than 20% throughout the MD simulation. Rg, SASA, and PSA were evaluated to assess the tightness, affinity, and ability to penetrate the cell membrane of the complex system. Rg is used to describe the size and shape distribution of a molecule or particle in three dimensions, which reflects how loose or tight the molecules are [31].The Rg values were found to be stable at 5–5.5 Å, which indicates that the binding of ligand–receptor was stable during the MD simulations. The SASA was basically below 100, and the PSA of compound **29** and C1 was around 120 Å, which suggests that they have some ability to penetrate through the cell membrane; nevertheless, the PSA of C2 and C3 was larger, which may enter the cell membrane through active transport. This is in agreement with the prediction of ADME, and taken together, C1 has superior pharmacokinetic properties.

The binding free energy between molecules can predict the binding modes between the target and drug, providing guidance for the subsequent optimization and modification of the drug. Binding free energy determination demonstrated that C2 exhibited the highest binding energy in IDH1, which was −93.25 ± 5.20 kcal/mol. It was found that three small molecules were better than compound **29**, which indicates that the binding of these three small molecules to the IDH1 protein is better than that of compound **29**. For drug design, hydrogen bonding analysis can help to optimize the binding of drugs to their target and improve the affinity and specificity of the drug [32]. Hydrogen bonding analysis displayed that ILE117, ILE130, and ASP275 formed stable hydrogen bonds with mIDH1 inhibitors. The results of this series of analyses provide theoretical guidance for the design of subsequent mIDH1 inhibitors.

Nowadays, there are many mIDH1 inhibitors in the research phase. However, only AG-120 has been approved by the FDA for marketing. A lot of designing of mIDH1 inhibitors is in the process of research, but there is a relative paucity of early theoretical research in terms of designing and synthesizing, leading to a lack of theoretical support during the process of synthesizing. The outcomes of biological activity research have been unsatisfactory. Therefore, we made an initial attempt to start with theoretical research using the methods of computer-aided drug design (CADD). Based on a series of derivatives with a pyridine-2-one skeleton that had already been designed, the 3D-QSAR model with good predictive ability was successfully constructed via known biological activities. Scaffold hopping was used to redesign and modify the parent structure from the point of QSAR. Using the most active compound **29** as the template molecule, part A was first transitioned. Interestingly, it was found that the docking score of the designed compounds was the best when the designed part A was pyridine-2-one, indicating that the introduction of piperazin-2-one at this position is beneficial. Following this, with the piperazin-2-one group in part A in our structural design, and part B though modified groups varying inhibitory activities were generated, providing a reliable theoretical basis for structural design. Furthermore, more in depth physicochemical properties, binding modes, and stabilities of the newly designed compounds were explored through MD simulations and ADME predictions. C1 was found to have better lipid solubility, easier passage through the lipid layer, better solubility, and higher saturation by ADME prediction. However, C2 and C3 had poorer pharmacological properties. MD simulations for evaluation of parameters like PSA, SASA also showed such results. The MM-GBSA results showed that the binding free energy of C1, C2, and C3 were all better than the positive control compound **29**, which indicates more stable binding and stronger affinity to IDH1 protein. However, in terms of drug-forming properties, parameters such as solubility and PSA of the molecules can be considered comprehensively, so that the drug candidates show higher activity, stronger potency, and longer-lasting action effects.

This study combined various methods of CADD, starting from construction of the model, prediction of the model, the design of small molecules, exploration of physicochemical properties, and comprehensive research on the binding modes, providing in depth guidance for the design and optimization of mIDH1 inhibitors. In the future, a series of derivatives designed based on this work will undergo chemical synthesis and biological activity validation.

## 4. Materials and Methods

### 4.1. Data Sets and Biological Activities

All 2H 1λ^2^ pyridin-2-ones 7,7-dimethyl-7,8-dihydro-2H-1λ^2^-quinoline-2,5(6H)-diones and their associated inhibitory activities were obtained from the literature [11]. The IC_50_ (μM) values of IDH1^R132H^ inhibitor activity were converted to their negative logarithmic (pIC_50_) values and used as dependent variables for the 3D-QSAR study. The 47 compounds were irregularly subdivided into a training set and a test set in a ratio of 4:1. Among them, the training set included 38 compounds and the test set included nine compounds.

### 4.2. Molecular Construction and Structure Optimization

The molecular structures of all compounds were drawn in ChemDraw 21.0 [33], and all molecular structures were optimized by MM2 in Chem3D 21.0. The module of “Minimize” in SYBYL-X 6.9 [34] based on Powell’s method was used to minimize small molecules. Gasteiger–Hückel charge was calculated under the tripos stance condition, the maximum number of iterations was set to 1000, the convergence threshold was set to 0.005. Through the module of Multisearch Option, for each compound about 200 conformations were obtained, and conformations with the lowest energy were selected for constructing the model. All other parameters were used as the system default values [28].

### 4.3. Molecular Alignment

Molecular alignment in respect of the common skeleton took into account one of the most significant ingredients in the process of building the model of 3D-QSAR [35]. Hence, the most active molecule, compound **29**, was employed for molecular alignment by fitting of atom-by-atom (Figure 3). After a common substructure was set, the dominant conformations of the remaining 46 compounds were selected for aligning.

### 4.4. Construction and Validation of 3D-QSAR Model

The model of 3D-QSAR was constructed by SYBYL-X 6.9. CoMFA [36] and CoMSIA [37] are the two most extensively used approaches of 3D-QSAR, which were used for analysis of the effect on molecular activity [38]. The Lennard–Jones and Coulomb potentials which represent the steric and electrostatic fields, were calculated as CoMFA descriptors using sp^3^ hybridized carbon probe atoms [39]. For the CoMSIA, the probe atom calculated the hydrogen bond acceptor field, hydrogen bond donor field, and hydrophobic field [40]. Partial least squares (PLS) is a multivariate statistical method for solving linear problems [41]. In addition, the attenuation coefficient α was set to 0.3. In CoMSIA, Gaussian distribution was introduced to assess the distance between the probe atom and each molecule [42].

The fields of CoMFA and CoMSIA were linearly correlated with the values of binding affinity via PLS analysis, with descriptors of CoMFA and CoMSIA used as independent variables and mIDH1 inhibitory activity (pIC_50_) as the dependent variable [43]. Cross-validation analysis was performed using the leave-one-out (LOO) to obtain the cross-validation correlation coefficient (q^2^) and the best group score (N). Then, a non-cross-validation analysis was performed based on N to obtain the correlation coefficient R^2^, standard error estimate (SEE), F-value, and contribution values of all fields. In addition, the prediction capacity of the elaborated models was investigated by determining the external validation coefficient (R^2^_pred_) that was computed using the equation. Non-cross-validation was performed to develop the final PLS model:

$$R_{prep}^{2}=1-\frac{PRESS}{SD}$$

where PRESS is the quadratic difference between the actual and estimated activity values of the compounds in the test set, and SD is the total of the squared deviations between the activity values in the test set and the mean activity values of the training set.

### 4.5. Scaffold Hopping and Virtual Screening 

The replace fragment of the receptor ligand interactions module of Discovery Studio 2020 [44] was used to perform scaffold hopping on the best biological activity compound **29**, where part A and the part B, respectively, were used for scaffold hopping. The input ligand was set to compound **29**, the structure of part A and the part B was partly selected as fragment for replacement, and all the databases were selected in the Fragment Libraries to pick the top 500. The Generate Fragment is false. Screening of small molecules needed to be minimized. all other values remained default. Finally, a total of 1000 small molecules were obtained by hopping part A and the part B, respectively. The SA score was calculated using RDKit [45].

To obtain the novel compounds with better docking scores, virtual screening-based docking was executed using the Virtual Screening Workflow of Schrödinger 2015 [46]. First of all, the compounds were prepared in the Ligprep module. The crystal structure of the IDH1 complex obtained from the RCSB protein data bank (PDB) was used for docking (PDB ID: 6B0Z). Subsequently, the crystal structure was pretreated via the module of the Protein Preparation Wizard in Schrödinger 2015, containing in addition to hydrogen and side chains, the elimination of water molecules, and the computation of partial charge and protonation via the force field of OPLS-2005. Then, though the module of Grid Generation in the Schrödinger 2015, a box was made to ascertain the binding pocket of IDH1. In the Virtual Screening Workflow module, compounds from scaffold hopping were used for the database in virtual screening, and then QikProp in the Filtering area was checked to run. Finally, the preparation of ligands was canceled in the screening process. Virtual screening used Schrödinger’s Glide, which is designed to assess the binding affinity of ligand to receptor. Herein, HTVS and SP as the criteria of evaluation were selected and the top 100 compounds were kept for subsequent screening at each step. Finally, the top 100 compounds had better docking scores than the positive control compound **29**. The rest of the values were left as default.

### 4.6. Prediction ADME

In order to ensure the pharmacokinetic properties of the candidates as well as the expected therapeutic effects in humans, the ADME of the top nine compounds was predicted through the SwissADME online server. ADME properties are an essential criterion for the assessment of drug-likeness [47]. An online prediction of ADME properties, SwissADME, was performed to explore the pharmacokinetic properties of compounds [48]. It was calculated by means of selecting the following parameters, LIPO, FLEX, INSATU, INSOLU, POLAR, SIZE and so on, to explore the pharmacokinetic properties of the top nine compounds.

### 4.7. Molecular Dynamics Simulations

To survey the dynamic stability of the screened compounds in the receptor, independent molecular dynamics simulations (three times) of the docked complex of IDH1 protein with the new top three designed compound with the best docking score and compound **29**, were carried out using the Desmond 2015 from Schrödinger, LLC (New York, NY, USA). Solvation was the first step of the dynamics where system builder was used for the same where the simple point charge (SPC) water model was employed in solvation [49]. An orthorhombic box was selected here, and neutralization was conducted of a fair number of sodium and chloride counter ions after calculations. The solvated system was then subjected to MD for 200 ns with the standard default protocol by opting for periodic boundary conditions with NPT ensemble (number of atoms, pressure, temperature). The temperature was adjusted to 310 K, pressure to 1.01325 atm and proceeded with MD. The rest of the parameters were left as default.

### 4.8. Trajectory Analysis

To monitor the binding stability of the protein–ligand complex during MD simulations, RMSD, RMSF, Rg, SASA, and PSA were employed for analyzing the trajectory [50]. The free energy landscape of protein folding on the IDH1 protein–ligand complex was measured using GROMACS 2020. The trajectory files obtained from Desmond were converted to the format of “trr” using VMD, and then the gmx tool in GROMACS 2020 was used to calculate FEL.

### 4.9. MM-GBSA

Binding free energies of the ligands towards the proteins were analyzed through the MM-GBSA method [51,52,53]. Different poses from the MD simulations for the complex were employed to estimate the binding affinity of protein–ligand and the stability of the complex [54].The trajectory files of 1000 frames were extracted from 3000–4000 frames in the last 50 ns. The ΔG_bind_ of the protein–ligand complexes was calculated via MM-GBSA [55].

## 5. Conclusions

In this study, a combined strategy of 3D-QSAR, scaffold hopping, ADME prediction, and MD simulations was used to explore the QSAR of pyridin-2-one and design a series of potentially effective inhibitors. The CoMFA (Q^2^ = 0.765, R^2^ = 0.980) and CoMSIA (Q^2^ = 0.770, R^2^ = 0.997) achieved decent results in respect of the statistical consequences. A series of compounds with novel structures was designed through scaffold hopping, and then 100 compounds with the best docking scores were identified through virtual screening. From 100 compounds, the top nine compounds with the best docking scores were selected for ADME analysis. It was found that C1, C3, and C5 had better predicted ADME description. Finally, MD simulations and binding free energy were performed for the top three compounds with the best docking scores. The backbones and ligands displayed as stable during the MD simulations of 200 ns. The result of the binding free energy suggested that C2 exhibited the highest binding free energy with IDH1, which is −93.25 ± 5.20 kcal/mol. This study explored more possibilities for CADD from a computational point of view using computer modeling, providing theoretical guidance for the design and synthesis of subsequent inhibitors.

## Figures and Tables

**Table 1:** Structure and corresponding activity data of reported mIDH1 inhibitors.

No.	R_1_	R_2_	R_3_	R_4_	R_5_	IC_50_(μM)	pIC_50_
**1**	Me	CH_3_	Et	H	Et	1.500	5.824
**2 ^a^**	Me	(CH_3_)_2_	Et	H	Et	1.200	5.920
**3**	Me	iPr	Et	H	Et	2.800	5.553
**4 ^a^**	Me	CH_2_CH_2_OH	Et	H	Et	0.420	6.380
**5**	Me	CH_2_CH_2_OMe	Et	H	Et	2.100	5.678
**6**	Me	CH_2_CO_2_H	Et	H	Et	1.200	5.921
**7**	Me	CH_2_CH_2_NH_2_	Et	H	Et	0.380	6.420
**8**	Me	CH_2_CH_2_NHMe	Et	H	Et	0.870	6.060
**9**	Me	CH_2_CONH_2_	Et	H	Et	1.600	5.796
**10**	Me	CH_2_CH_2_CH_2_NHMe	Et	H	Et	1.000	6.000
**11**	Me	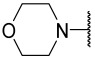	Et	H	Et	4.200	5.377
**12 ^a^**	Me	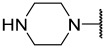	Et	H	Et	0.210	6.680
**13 ^a^**	Me	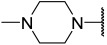	Et	H	Et	2.900	5.540
**14**	nPr	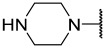	Et	H	Et	0.075	7.125
**15**	iPr	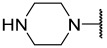	Et	H	Et	0.420	6.377
**16**	iBu	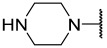	Et	H	Et	0.110	6.959
**17 ^a^**	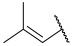	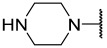	Et	H	Et	0.044	7.360
**18**	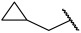	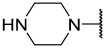	Et	H	Et	0.055	7.260
**19**	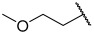	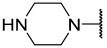	Et	H	Et	0.140	6.854
**20**	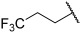	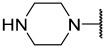	Et	H	Et	0.240	6.620
**21**	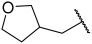	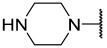	Et	H	Et	0.075	7.125
**22**	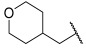	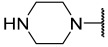	Et	H	Et	1.100	5.959
**23**	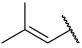	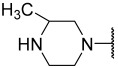	Et	H	Et	0.044	7.357
**24**	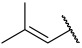	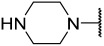	Et	H	Et	0.039	7.409
**25**	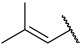	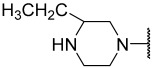	Et	H	Et	0.100	7.000
**26**	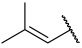	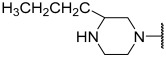	Et	H	Et	0.130	6.886
**27**	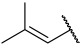	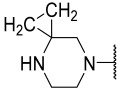	Et	H	Et	0.078	7.108
**28**	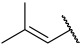	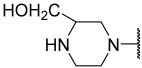	Et	H	Et	0.049	7.310
**29**	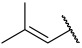	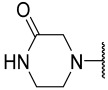	Et	H	Et	0.035	7.456
**30**	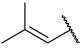	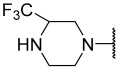	Et	H	Et	0.360	6.444
**31**	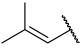	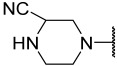	Et	H	Et	0.160	6.796
**32**	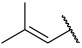	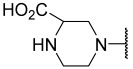	Et	H	Et	0.055	7.260
**33**	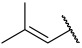	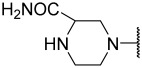	Et	H	Et	0.094	7.027
**34**	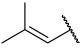	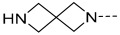	Et	H	Et	0.240	6.620
**35 ^a^**	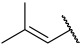	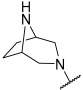	Et	H	Et	0.094	7.030
**36 ^a^**	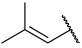	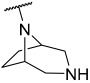	Et	H	Et	0.062	7.210
**37 ^a^**	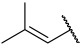	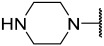	H	H	H	0.330	6.480
**38**	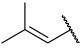	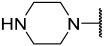	Et	H	H	0.056	7.252
**39**	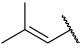	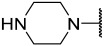	Et	H	Me	0.270	6.569
**40**	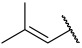	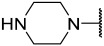	Et	H	Cl	0.044	7.357
**41**	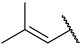	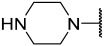	Et	Me	H	0.044	7.357
**42 ^a^**	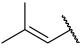	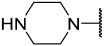	Et	OMe	H	0.114	6.940
**42**(+)	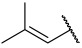	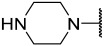	Et	OMe	H	0.120	6.921
**42**(-)	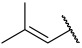	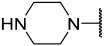	Et	OMe	H	0.420	6.377
**43**	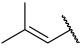	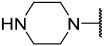	Et	Cl	H	0.114	6.943
**43**(+)	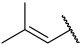	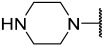	Et	Cl	H	0.081	7.092
**43**(-)	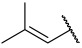	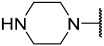	Et	Cl	H	0.250	6.602

^a^ Test set for the 3D-QSAR model.

**Table 2:** PLS statistical results of CoMSIA models in different molecular field combinations.

3D-OSAR Models	R^2^	Q^2^	F	SEE	N	Fraction				
						Steric	Electrostatic	Hydrophobic	Donor	Acceptor
CoMSIA/SE	0.968	0.691	158.496	0.115	6	0.372	0.628			
CoMSIA/SH	0.926	0.794	102.908	0.171	4	0.406		0.594		
COMSIA/SA	0.945	0.598	88.140	0.152	6	0.546				0.454
CoMSIA/EH	0.973	0.673	185.305	0.106	6		0.566	0.434		
CoMSIA/EHA	0.986	0.626	296.797	0.078	7		0.404	0.308		0.287
CoMSIA/SEA	0.981	0.652	261.932	0.090	6	0.256	0.452			0.292
CoMSIA/SEH	0.997	0.770	800.063	0.040	10	0.225	0.444	0.331		
CoMSIA/SHDA	0.954	0.596	172.339	0.134	4	0.103		0.142	0.500	0.255
CoMSIA/SEHD	0.998	0.669	1286.243	0.033	9	0.116	0.249	0.149	0.485	
CoMSIA/SEHA	0.983	0.710	307.710	0.083	6	0.175	0.326	0.249		0.250
CoMSIA/SEHDA	0.991	0.638	494.747	0.061	7	0.084	0.186	0.118	0.417	0.195

**Table 3:** Summary of CoMFA and CoMSIA models.

PLS Statistics	CoMFA	CoMSIA
Q^2^	0.765	0.77
N	6	10
R^2^	0.980	0.997
R^2^_pred_	0.943	0.980
F	253.629	800.063
SEE	0.091	0.04
Steric	0.582	0.225
Electrostatic	0.418	0.444
Hydrophobic	-	0.331

**Table 4:** Experimental pIC_50_ (Exp.), predicted pIC_50_ (Pred.), and corresponding residuals (Res.) of the pyridin-2-one derivatives.

No.	pIC_50_	CoMFA		CoMSIA	
	Exp.	Pred.	Res.	Pred.	Res.
**1**	5.824	5.821	0.003	5.785	0.039
**2 ^a^**	5.920	6.056	−0.136	6.025	−0.105
**3**	5.553	5.631	−0.078	5.549	0.004
**4 ^a^**	6.380	6.394	−0.014	6.358	0.022
**5**	5.678	5.603	0.075	5.682	−0.004
**6**	5.921	5.907	0.014	5.935	−0.014
**7**	6.420	6.369	0.051	6.412	0.008
**8**	6.060	6.007	0.053	6.118	−0.058
**9**	5.796	5.780	0.016	5.779	0.017
**10**	6.000	5.999	0.001	5.977	0.023
**11**	5.377	5.566	−0.189	5.423	−0.046
**12 ^a^**	6.680	6.677	0.003	6.608	0.072
**13 ^a^**	5.540	5.525	0.015	5.536	0.004
**14**	7.125	7.152	−0.027	7.149	−0.024
**15**	6.377	6.181	0.196	6.337	0.04
**16**	6.959	7.037	−0.078	6.947	0.012
**17 ^a^**	7.360	6.567	0.793	7.055	0.305
**18**	7.260	7.145	0.115	7.222	0.038
**19**	6.854	6.883	−0.029	6.909	−0.055
**20**	6.620	6.603	0.017	6.578	0.042
**21**	7.125	7.156	−0.031	7.113	0.012
**22**	5.959	6.001	−0.042	5.982	−0.023
**23**	7.357	7.322	0.035	7.34	0.017
**24**	7.409	7.535	−0.126	7.415	−0.006
**25**	7.000	7.017	−0.017	7.011	−0.011
**26**	6.886	6.906	−0.02	6.911	−0.025
**27**	7.108	7.161	−0.053	7.17	−0.062
**28**	7.310	7.392	−0.082	7.295	0.015
**29**	7.456	7.510	−0.054	7.492	−0.036
**30**	6.444	6.573	−0.129	6.411	0.033
**31**	6.796	6.824	−0.028	6.786	0.010
**32**	7.260	7.124	0.136	7.262	−0.002
**33**	7.027	7.030	−0.003	7.050	−0.023
**34**	6.620	6.659	−0.039	6.638	−0.018
**35 ^a^**	7.030	7.031	−0.001	7.052	−0.022
**36 ^a^**	7.210	7.252	−0.042	7.372	−0.162
**37 ^a^**	6.480	6.481	−0.001	6.856	−0.376
**38**	7.252	7.121	0.131	7.200	0.052
**39**	6.569	6.559	0.010	6.565	0.004
**40**	7.357	7.28	0.077	7.372	−0.015
**41**	7.357	7.348	0.009	7.319	0.038
**42 ^a^**	6.940	6.959	−0.019	6.957	−0.017
**42**(+)	6.921	6.991	−0.070	6.922	−0.001
**42**(-)	6.377	6.430	−0.053	6.365	0.012
**43**	6.943	7.008	−0.065	7.020	−0.077
**43**(+)	7.092	6.920	0.172	7.008	0.084
**43**(-)	6.602	6.500	0.102	6.601	0.001

^a^ Test set for the 3D-QSAR model.

**Table 5:** Chemical structure, docking scores, predictive activity value, SA scores of the top nine compounds.

No.	Structure	Docking Score (Kcal/mol)	CoMFA	CoMSIA	SA Scores
C1	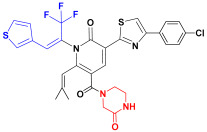	−11.865	7.036	6.874	3.254
C2	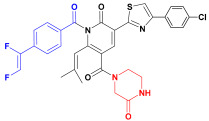	−11.575	6.107	6.115	3.172
C3	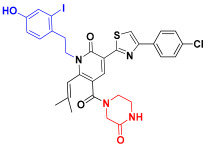	−11.536	7.289	7.524	3.181
C4	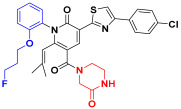	−11.304	6.617	6.486	3.694
C5	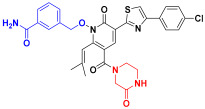	−11.277	6.746	6.954	3.474
C6	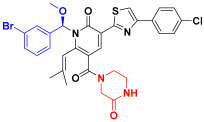	−11.146	7.346	7.374	3.477
C7	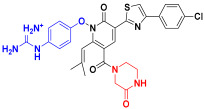	−11.110	6.838	6.694	3.485
C8	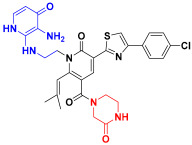	−11.085	6.463	9.130	3.511
C9	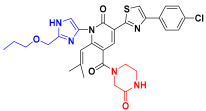	−11.076	7.132	7.348	3.352
Compound **29**	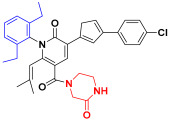	−9.450	7.510	7.492	3.385

**Table 6:** Binding free energy calculation of the molecule through MM-GBSA.

No.	Structure	MM-GBSA ΔG_bind_ (kcal/mol)
Compound **29**	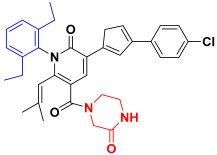	−75.54 ± 5.69
C1	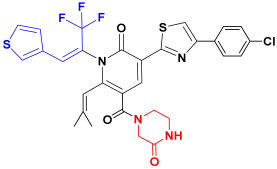	−76.34 ± 4.67
C2	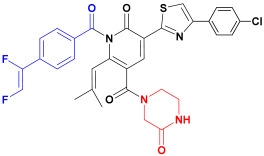	−93.25 ± 5.20
C3	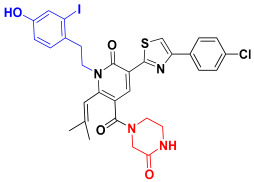	−80.60 ± 4.29

## Data Availability

All relevant data are included in the manuscript.
